# The Flexural Strength and Fracture Toughness of TC4-Based Laminated Composites Reinforced with Ti Aluminide and Carbide

**DOI:** 10.3390/ma10101175

**Published:** 2017-10-13

**Authors:** Yanhan Fei, Taotao Ai, Qunfei Niu, Wenhu Li, Xinqiang Yuan, Ran Jing, Hongfeng Dong

**Affiliations:** 1 School of Materials Science and Engineering, Shaanxi University of Technology, Hanzhong 723000, China; sky_snowstorm@163.com (Y.F.); niu119257289@163.com (Q.N.); mse_liwh@snut.edu.cn (W.L.); yxq_hb@126.com (X.Y.); qwe_jr@163.com (R.J.); hfdong@hotmail.com (H.D.); 2 Shaanxi Key Laboratory of Industrial Automation, Hanzhong 723000, China

**Keywords:** laminated composite materials, mechanical properties, microstructure, spark plasma sintering (SPS)

## Abstract

TiC–Ti–Al mixed powders and TC4 titanium alloy foils were overlapped layer-by-layer in the graphite die. The TC4-based laminated composite sheets reinforced by Ti aluminide and carbide were successfully fabricated via spark plasma sintering (SPS) at 1100 °C with a well-bonded interface. The composite layers were mainly composed of TiAl, Ti_3_Al, Ti_2_AlC, and Ti_3_AlC_2_ phases. The carbides particles distributed in the matrix played an important role in the deflection of cracks and the passivation of microcracks. TC4 titanium alloy layers had an obvious effect on the stress distribution during the loading process, and provided an energy dissipation mechanism, which could improve the mechanical properties of the laminated composite sheets obviously. When the theoretical amount of Ti_2_AlC was 20 wt %, the flexural strength and fracture toughness of the laminated composite sheets reached the maximum value in the arrester direction, which were 1428.79 MPa and 64.08 MPa·m^1/2^, respectively.

## 1. Introduction

TiAl-based alloys have properties such as low density, a high melting point and elastic modulus, favorable corrosion and oxidation resistance, good environmental stability, and flame resistance. Their working temperature can reach 750–900 °C, which is close to that of the widely used Ni-based alloys, but the density is only half of the latter. Therefore, TiAl-based alloys have the potential to replace Ni-based alloys as aerospace structural components and transmission parts of power systems, which can greatly improve the thrust-weight ratio and fuel efficiency of the engines [1,2,3,4,5]. However, the intrinsic brittleness results in poor processing performance; thus, it hinders their further applications. Shells naturally have a high strength and toughness, with particular structures that inspire us [6]. We have designed TiAl-based laminated composite sheets with shell-like composite structures; they can be prepared by overlapping the high-strength brittle layer and the high-toughness layer alternately, and this is an effective method of optimizing the overall performance of the metals or alloys. We selected a TC4 titanium alloy (Ti–6Al–4V) as the toughening layer, which has the advantages of low density (4.5 kg/m^3^), high melting point (1660 °C), good corrosion resistance, and high specific strength and plasticity [7]. In recent years, the ternary carbides and nitrides with the general formula M*_n_*_+1_AX*_n_* (abbreviated as MAX) represent a new class of solids, where *n* = 1, 2, or 3; M represents an early transition metal; A is an A-group element (a subset of group 13–16 elements); and X is C and/or N element [8,9,10]. MAX compounds have a combination of excellent performance of both metals and ceramics, which have become research hotspots. Among them, the thermal expansion coefficient of Ti_2_AlC is close to TiAl, which is considered to be one of the ideal reinforcements for TiAl-based alloys. Recently, there are many studies that focus on Ti_2_AlC-reinforced TiAl-based alloys [11,12,13,14].

Our study combines the concept of biomimetic laminated structure design and particle enhancement technology. In this paper, using TC4 titanium alloy foils as toughening layers, these foils and TiC–Ti–Al mixed powders were overlapped layer-by-layer in the die. The TiAl-based laminated composite sheets co-reinforced by titanium alloy layers and MAX particles were then prepared via spark plasma sintering (SPS). The microstructure and mechanical properties of the composite sheets were studied, and the strengthening and toughening mechanisms are discussed. SPS technology is shown to achieve rapid sintering and densification of the powders at a relatively low temperature and short time, which can effectively restrain the diffusion reaction between Ti and Al, so that the TC4 titanium alloy layers as the toughening layers can be well preserved.

## 2. Materials and Methods

Commercial TiC powders (purity ≥ 99.5%, average particle size < 20 μm), Ti powders (purity ≥ 99.5%, average particle size < 35 μm) and Al powders (purity ≥ 99.5%, average particle size < 55 μm) were used as the raw powdered materials. TC4 titanium alloy foils served as the toughening layers, and the number of the layers was selected to be 9. Ti–Al–TiC mixed powders served as the composites layers, and according to the formula (1 + *n*)Ti + (1 + *n*)Al + TiC = *n*TiAl + Ti_2_AlC, the content of Ti, Al, and TiC was calculated while the theoretical amount of Ti_2_AlC was 5 wt %, 10 wt %, 20 wt %, and 30 wt %, respectively. For comparison, Ti–48Al (atom %) mixed powders were used to prepare the laminated sheets without MAX reinforcements. The formula is shown in Table 1. 

**Figure 1:**
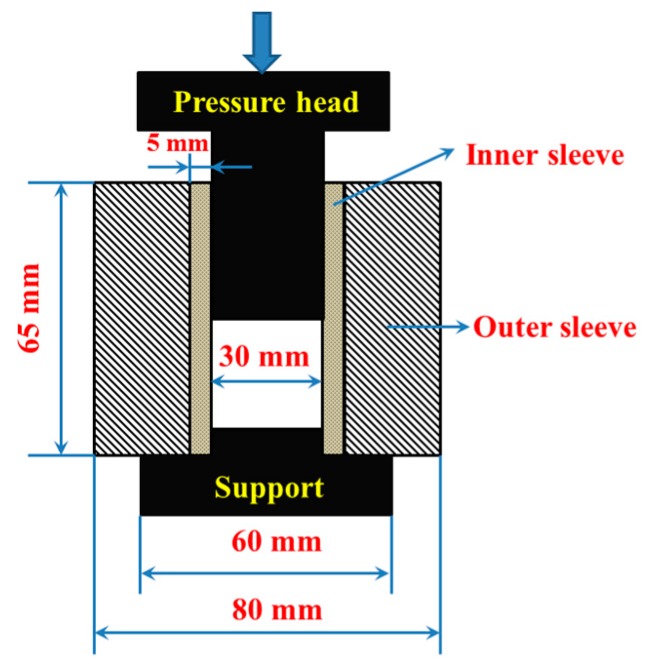
The size of the graphite die.

**Figure 2:**
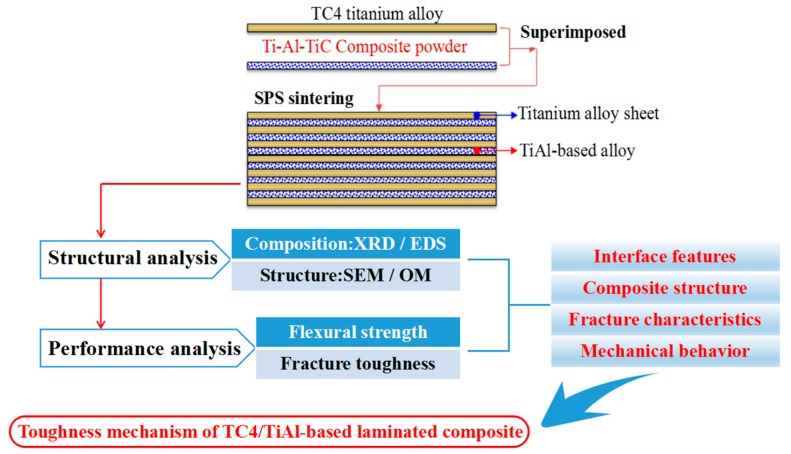
The flow diagram of the experiment.

The Ti–48Al (atom %) and Ti–Al–TiC powders were ball-milled for 4 h with a milling ratio of 180 rpm in a planetary ball grinder with stainless steel containers and balls, and then passed through a 200 mesh sieve. The surface of the TC4 titanium alloy (Ti–6Al–4V) foils with a diameter of 30 mm and a thickness of 0.3 mm was roughed with steel brushes at first. The foils were then washed with a 10 wt % HF solution to remove the oxides on the surface. Finally, they were washed with distilled water and acetone before vacuum drying. After that, the Ti–Al–TiC mixed powders and TC4 titanium alloy foils were overlapped layer-by-layer in the graphite die and cold-pressed using a pressure of 15 MPa. The size of the graphite die is shown in Figure 1. The green bodies were sintered via a spark plasma sintering (SPS, SPS-20T-10, Shanghai Chenhua, Shanghai, China) system under vacuum (<10^−2^ Pa) to obtain the laminated composite sheets. From room temperature to 900 °C, the temperature rate was 50 °C/min. Above 900 °C, the temperature rate was 200 °C/min. The samples were then sintered at 1100 °C for 3 min under a pressure of 30 MPa. Finally, the samples were cooled down to room temperature. The thickness of the as-sintered samples was 6 mm. Figure 2 shows the flow diagram of the experiment in this paper.

The phase identification of the as-obtained laminated composite sheets was performed with an X-ray diffractometer (XRD, D/max-2200PC, Tokyo, Japan). The microstructure of the specimens was investigated by scanning electron microscopy (SEM, JSM-6700F, Tokyo, Japan) coupled with an energy-dispersive spectroscopy (EDS) for chemical analysis. The polished surface of the products was etched in a reagent of 5 vol % HF and 95 vol % H_2_O before observing the microstructure in optical microscopy (OM, PMG-3, Olympus, Ishikawa, Japan).

The test sample size was 25 mm (length) × 4 mm (width) × 4 mm (height). A three-point bending test was performed using a span of 20 mm with a crossing speed of 5 mm/min to measure flexural strength. We measured three samples to obtain an average value. The flexural strength of the samples is calculated from the fracture or failure load. The calculation formula of the flexural strength is

(1) $$\sigma_{b}=\frac{3PL}{2bh^{2}}$$

where *σ_b_* is strength (MPa), *P* is the maximum load (N) when the specimen breaks or fails, *L* is span (mm), *b* is width (mm), and *h* is specimen height (mm).

**Figure 3:**
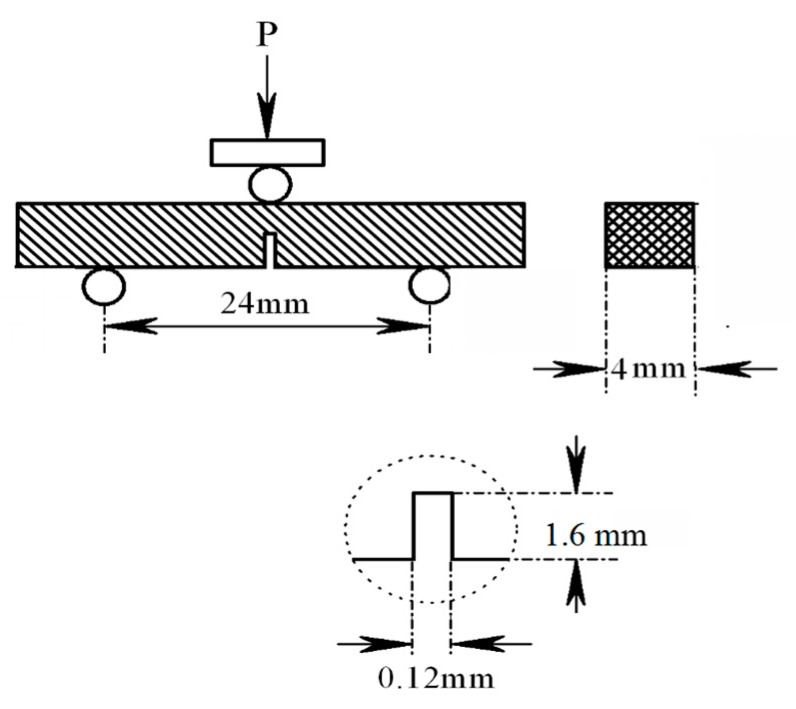
Schematic representation of the sample geometry for fracture toughness test.

The fracture toughness was measured using single-edge notched beam (SENB) method with a crosshead speed of 0.05 mm/min and a span of 24 mm at room temperature in air, according to the ASTM: E399-90 test standard [15]. The SENB specimens were machined via electrical discharge machining (EDM, TI-40S, ZhongXing CNC Machine, Taizhou, China). A notch with a depth of 0.4 W (where W is the width of the specimen) and a width of 0.12 mm was made. We measured three samples to obtain the average value of the fracture toughness. The specific geometric model of the bend specimen is shown in Figure 3. The calculation formula of the fracture toughness is [15]

*K* = (*PS*/*BW*^3/2^) × *f* (*a*/*W*) (2)

where *P* is the load, *S* is the span, *B* is the specimen thickness, *W* is the specimen width, a is the crack length, and *f* (*a*/*W*) is the function defined as [15]

(3) $$f(a/W)=\frac{3{\left(a/W\right)}^{1/2}[1.99-(a/W)(1-a/W)\times (2.15-3.93a/W+2.7{\left(a/W\right)}^{2})]}{2(1+2a/W){\left(1-a/W\right)}^{3/2}}$$

## 3. Results and Discussion

**Figure 4:**
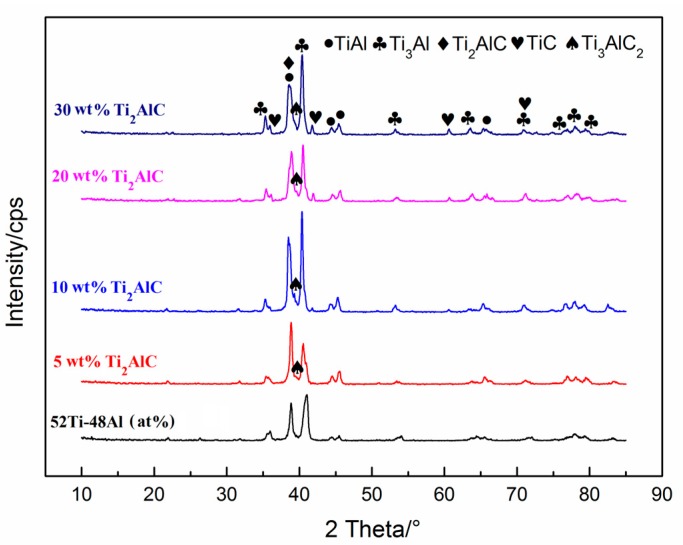
XRD patterns of the laminated composite sheets with different theoretical amounts of Ti_2_AlC.

Figure 4 shows the XRD patterns of the laminated composite sheets with different theoretical amounts of Ti_2_AlC on the cross section of the specimens. It can be seen in Figure 4 that the product of Ti–48Al (atom %) mixed powders after sintering at 1100 °C is mainly composed of TiAl and Ti_3_Al phases. With the addition of TiC, the products of TiC–Ti–Al mixed powders after sintered at 1100 °C are mainly made up of TiAl and Ti_3_Al, Ti_2_AlC, Ti_3_AlC_2_, and a small amount of TiC. With the increase in TiC content, the theoretical amount of Ti_2_AlC gradually increases, and Ti_3_AlC_2_ content obviously decreases, which may be caused by the decomposition of Ti_3_AlC_2_.

Thus, the possible reactions during the sintering process are summarized as follows:

Ti + 3Al → TiAl_3_ (4)

TiAl_3_ + 4T → 2TiAl + Ti_3_Al (5)

TiAl + TiC → Ti_2_AlC (6)

Ti_2_AlC + TiC → Ti_3_AlC_2_ (7)

Ti_3_AlC_2_ → Ti_2_AlC + TiC. (8)

**Figure 5:**
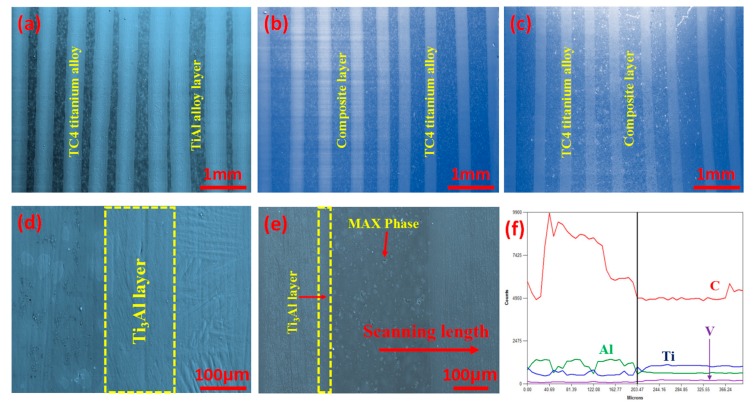
The SEM images of the surface and the EDS line scanning image of the laminated composite sheets. (**a**,**d**) 0 wt % Ti_2_AlC; (**b**,**e**) 5 wt % Ti_2_AlC; (**c**) 10 wt % Ti_2_AlC; (**f**) EDS line scanning image.

Figure 5 shows the SEM images of the surface and the EDS line scanning image of the laminated composite sheets. It can be seen in Figure 5a,d that Ti_3_Al interface layers with a thickness of 150 μm are formed in the composite sheet without Ti_2_AlC due to the intense interface reaction, and the thickness of the TiAl alloy layers is relatively thinner and uneven. It can be seen from Figure 5b,c that, after doping with TiC, the thickness of the TC4 titanium alloy layers is 350 μm after the SPS process, indicating that the TC4 titanium alloy foils reacted with the Ti–Al–TiC mixed powders during the SPS process. The interface layers are thinner than that of the product without Ti_2_AlC, indicating that TiC can restrain the interface reaction. However, the thickness is higher than that of the TC4 titanium alloy foils. The thickness of Ti_2_AlC/TiAl composite layers is about 500–700 μm. Figure 5e shows that the composite layers and the TC4 titanium alloy toughening layers are well bonded without macroscopic and microscopic cracks, and the thickness of the interface layers is about 20–50 μm. Figure 5f shows the EDS line scanning image of the laminated composite sheet corresponding to Figure 5e. The change of Ti and Al proves that the Ti_3_Al interface layers are formed between the toughening layers and the composite layers.

**Figure 6:**
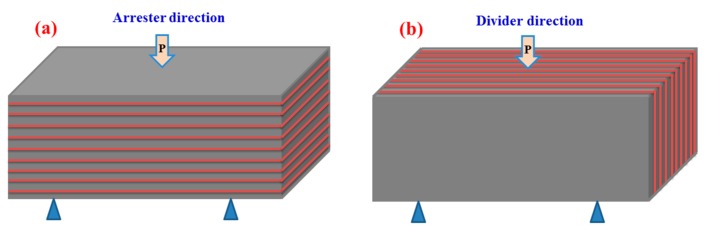
The testing directions for the fracture toughness test. (**a**) Arrester direction; (**b**) Divider direction.

Due to the particular laminated structure of the composite sheets, the performance tests were employed in different directions as shown in Figure 6.

**Figure 7:**
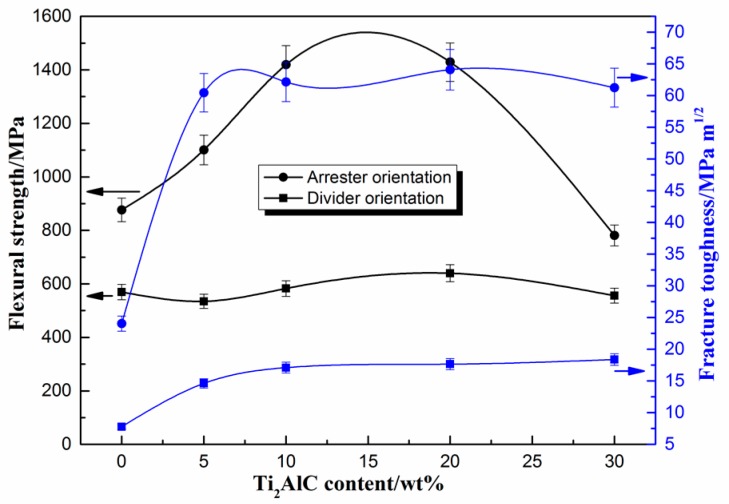
The flexural strength and fracture toughness of the laminated composite sheets in different loading directions.

**Figure 8:**
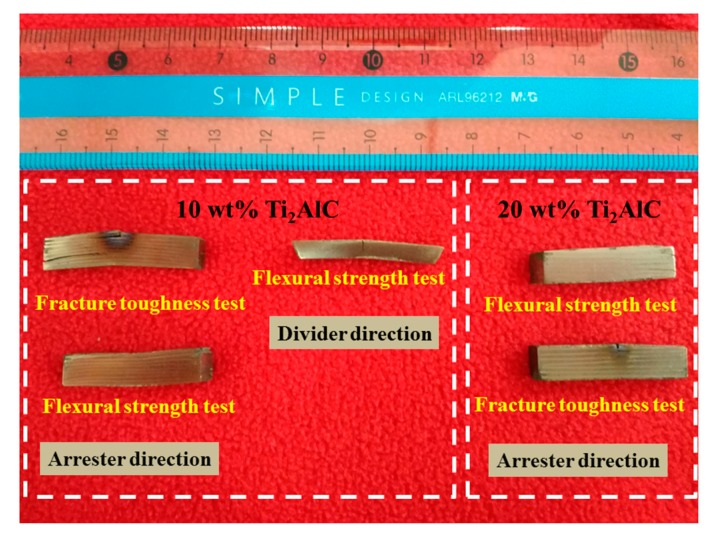
The specimens after the mechanical properties tests on different loading directions.

Figure 7 shows the flexural strength and fracture toughness of the laminated composite sheets in the arrester direction and in the divider direction. As seen in Figure 7, the mechanical properties of the laminated composite sheets exhibit anisotropy. In the arrester direction, the flexural strength and fracture toughness of the laminated sheets without Ti_2_AlC are 876.41 MPa and 24.04 MPa·m^1/2^, respectively. With the increase in the theoretical amount of Ti_2_AlC, the flexural strength and fracture toughness increase, and when the concentration reaches 20 wt %, the flexural strength and fracture toughness reach maximum values, which are 1428.79 MPa and 64.08 MPa·m^1/2^, respectively. The flexural strength and fracture toughness are 63.0% and 166.6% higher than that of the TC4/TiAl laminated sheets. When the theoretical amount of Ti_2_AlC increases further, the flexural strength and fracture toughness decreases appropriately. Compared to the (TiB/Ti)-Ti_3_Al laminated composite [16], the maximum flexural strength and fracture toughness in the arrester direction are 122.9% and 148.4% higher than its values. Compared to Ti_3_AlC_2_-Ti_2_AlC/TiAl composites [17], the maximum flexural strength and fracture toughness rose 352.2% and 777.8%, respectively. Compared to Al_2_O_3_/TiAl composites [18], the maximum flexural strength and fracture toughness rose 54.5% and 649.5%. In the divider direction, the flexural strength and fracture toughness of the laminated sheet without Ti_2_AlC are 569.58 MPa and 7.78 MPa·m^1/2^, respectively. With the increase in the theoretical amount of Ti_2_AlC, the flexural strength and fracture toughness rise. When the Ti_2_AlC concentration is 20 wt %, the flexural strength reaches a maximum value of 639.77 MPa. When the Ti_2_AlC concentration is 30 wt %, the fracture toughness achieves a maximum value of 18.39 MPa·m^1/2^. Table 2 is a comparison of the test results of mechanical properties in this paper with these of other researches. Compared to the composite [16], the maximum flexural strength in the divider direction enhances by 21.4%, while the fracture toughness slightly decreases. However, the strength and toughness still significantly improve, compared to TiAl alloys [17,18]. Figure 8 shows the specimens after the mechanical properties tests on different loading directions. It can be seen clearly that the specimens do not fracture after the mechanical properties tests. This indicates that the composite sheets have good performance.

As shown in Figure 7, with the same theoretical amount of Ti_2_AlC, the flexural strength and fracture toughness of the laminated composite sheets in the arrester direction are greatly higher than those in the divider direction. With the increase in the amount of Ti_2_AlC, the mechanical properties of the sheets in the divider direction slightly change, indicating that the improvement of the mechanical properties is mainly dependent on the energy dissipation mechanism of the laminated structures. To regard the sheet as a closed system, in the arrester stress direction, the entropy increase of the whole system is layer-by-layer dispersed into each layer by means of the energy transmit between the layers [6]. As a result, the entropy of each layer structure has a limited increase. However, in the divider stress direction, the entropy increase of the whole system cannot effectively participate, assembles in the stress zone, and thus surpasses the critical value easily and quickly, ultimately causing damage and simultaneously conducting heat exchange with the outside [19]. Therefore, utilizing the energy dissipation mechanism reasonably, the flexural strength and fracture toughness of the laminated composite sheets at room temperature can be greatly improved.

**Figure 9:**
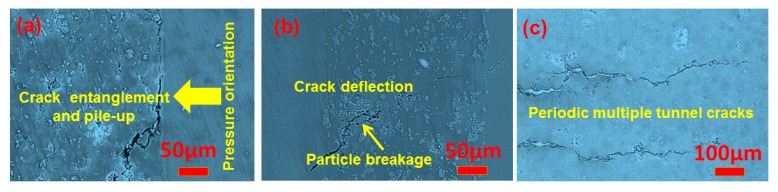
The crack propagation of the laminated composite sheets corresponding to 20 wt % Ti_2_AlC theoretical amount. (**a**) Crack entanglement and pile-up; (**b**) Tip passivation behavior of the crack at the tough layer; (**c**) Periodic multiple tunnel cracks.

Figure 9 is the crack propagation of the laminated composite sheets corresponding to a 20 wt % Ti_2_AlC theoretical amount. As seen in Figure 9a, near the load side of the sheet, cracks extend along the bonding interface. The cracks deflect into the composite layers when extending some distance (Figure 9b). The existence of second phases can hinder crack propagation and lead to a decrease in the crack extending width; the crack driving force in this case gradually decreases [19]. In Figure 9c, periodic multiple tunnel cracks appear in the products, which can improve the mechanical properties further. No macrocracks or any cracking phenomenon is observed on the far side of the load. As shown in Figure 9a,b, the crack-tip stress fields generate tangling so that the the crack-tip stress fields can’t be decoupled in the process of crack propagation and then resulted to the distortion of stress fields. The distortion of stress fields finally aroused the stress concentration which is the main driving force of crack propagation. It can be seen in Figure 9a,b that, when the cracks in the composite material layer extend to the toughening layer, it mainly propagates along the bonding interface. Due to the well bonded interface, the crack width gradually decreases, indicating that the crack driving force is reduced and the crack tip is passivated [20,21,22,23]. Thus, the crack propagation is obviously hindered. In addition, it is observed that, when the crack in the arrester direction extends from the brittle layer to the toughening layer, it does not completely cross the toughening layer, indicating that the TC4 titanium alloy layers have a good toughening effect, leading to a significant improvement in fracture toughness.

**Figure 10:**
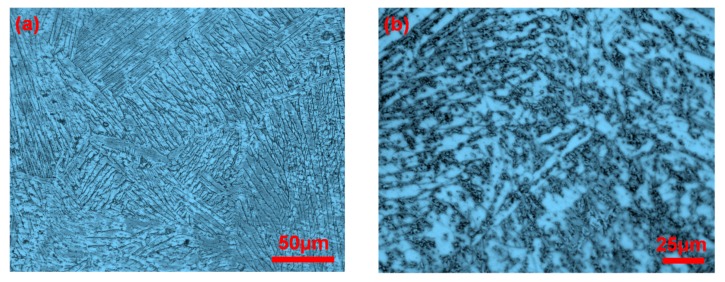
The OM images on composite layers of the laminated composite sheets with different Ti_2_AlC theoretical concentrations: (**a**) 5 wt % Ti_2_AlC; (**b**) 30 wt % Ti_2_AlC.

In addition, the fine in situ carbides particles play a role in particle strengthening and significantly influence the mechanical properties of the laminated composite sheets. Figure 10 is the OM images of the composite layers of the laminated composite sheets with different Ti_2_AlC theoretical concentrations. It can be seen clearly that the carbides are mainly distributed in the grain boundaries. It can also be seen that the content of the carbides increases with the increase in the amount of TiC doping. Carbides pinned at the TiAl grain boundaries hinder the growth of the γ/α_2_ grains and obviously refine the grains, which can restrain crack propagation. The carbides can interact with the dislocations in the grains to further intensify the strengthening effect of the MAX phases. However, when the theoretical concentration of Ti_2_AlC increases to 30 wt %, the carbides agglomerate, as shown in Figure 10b, which will reduce the mechanical properties of the material. In Figure 9b,c, crack branching, crack deflection, MAX particle breaking, and periodic double-crack propagation can be observed in the product. These mechanical behaviors mentioned above will consume a large amount of energy in the process of crack propagation and thus weaken the driving force of the crack extension, leading to a substantial improvement in the flexural strength and fracture toughness. As seen in Figure 7, it can also be found that the flexural strength and fracture toughness of the laminated composite sheet with Ti_2_AlC are better than that of the laminated sheet without Ti_2_AlC in the arrester and divider directions. However, when the amount of Ti_2_AlC increases to 30 wt %, the flexural strength in the arrester direction is only 780.95 MPa, which decreases by 45.3% compared to the product with 20 wt % Ti_2_AlC. This indicated that the proper content of MAX phases has an important effect on the mechanical properties of the laminated composite sheets.

**Figure 11:**
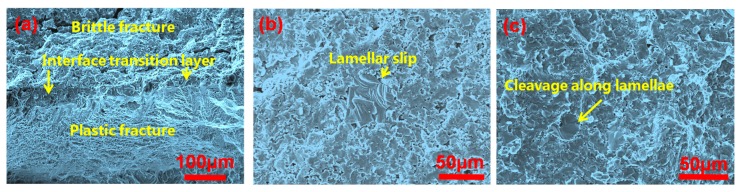
Fracture SEM images of the laminated composite sheets corresponding to 20 wt % Ti_2_AlC theoretical amount. (**a**) Fracture SEM image of laminated structure composites at interface; (**b**) SEM image at the fracture of composite layer; (**c**) Mechanical behavior of cleavage along the lamellae in fracture.

Figure 11 shows the fracture SEM images of the laminated composite sheets after bend tests in the arrester direction with 10 wt % Ti_2_AlC. In Figure 11, the fracture of the composite layer shows brittle fracture characteristics; for example, cleavage fracture appears in the product. For the γ/α_2_ titanium aluminum alloy, the microcrack initiation mechanism strongly depends on the orientation of the lamellar structure and the force direction [24]. When the loading axis is perpendicular to the lamellar structure, the microcracks mainly focus on the γ/α_2_ crystal face. As shown in Figure 11c, we can see the slip of the lamellar structure. Without TiC doping, the γ/α_2_ grains are coarse and the crack propagation resistance is very weak, so the crack propagation rate is fast. After doping with TiC, the composite layer presents fine lamellar microstructure due to the pinning of the MAX phases, so there are more γ/α_2_ crystal faces in the same stress section. Plugging, entanglement, and other mechanical behaviors that occur in the microcrack propagating process obviously hinder crack propagation. For the toughening layers of the TC4 titanium alloys, the fracture mode presents a plastic fracture. A different strengthening mechanism of the laminated composite sheets in the fracture process can significantly improve the mechanical properties of the materials.

## 4. Conclusions

(1) TiC–Ti–Al mixed powders and TC4 titanium alloy foils were overlapped layer-by-layer in the graphite die. The laminated TiAl-based composite sheets were successfully fabricated via spark plasma sintering (SPS) at 1100 °C. The laminated composite sheets were mainly composed of TiAl, Ti_3_Al, Ti_3_AlC_2_, Ti_2_AlC, and a small amount of TiC phases. The composite layer and the TC4 titanium alloy layer were well bonded.
(2) The mechanical properties of the laminated composite sheets exhibit anisotropy. The flexural strength and fracture toughness of the laminated composite sheets in the arrester direction are much higher than those in the divider direction. When the theoretical amount of Ti_2_AlC is 20 wt %, the flexural strength and fracture toughness of the laminated composite sheets in the arrester direction reach maximum values of 1428.79 MPa and 64.08 MPa·m^1/2^, which are 63.0% and 166.6% higher than that of the TC4/TiAl laminated sheets. The carbides particles distributed in the matrix play an important role in the deflection of cracks and the passivation of microcracks. TC4 titanium alloy layers have an obvious effect on the stress distribution during the loading process and exert an energy dissipation mechanism that can obviously improve the mechanical properties of the laminated composite sheets.

## Figures and Tables

**Table 1:** Formula of the laminated composite sheets.

Theoretical Content of Ti_2_AlC (wt %)	Formula			The Number of TC4 Sheets
	Ti (wt %)	Al (wt %)	TiC (wt %)	
0	63.84	36.16	0	9
5	61.81	34.83	3.36	
10	59.77	33.69	6.54	
20	56.01	31.57	12.42	
30	52.58	29.64	17.78	

**Table 2:** The mechanical properties of some materials.

Materials	Flexural Strength/MPa		Fracture Toughness/MPa·m^1/2^	
	Arrester	Divider	Arrester	Divider
TC4/TiAl laminated composite	876.41	569.58	24.04	7.78
TC4/20 wt % Ti_2_AlC-TiAl laminated composite	1428.79	639.77	64.08	17.66
TC4/30 wt % Ti_2_AlC-TiAl laminated composite	780.95	555.84	61.25	18.39
(TiB/Ti)-Ti_3_Al laminated composite [16]	641	527	25.8	21.4
Ti_3_AlC_2_–Ti_2_AlC/TiAl Composite [17]	316	316	7.3	7.3
Al_2_O_3_/TiAl composite [18]	925	925	8.55	8.55
